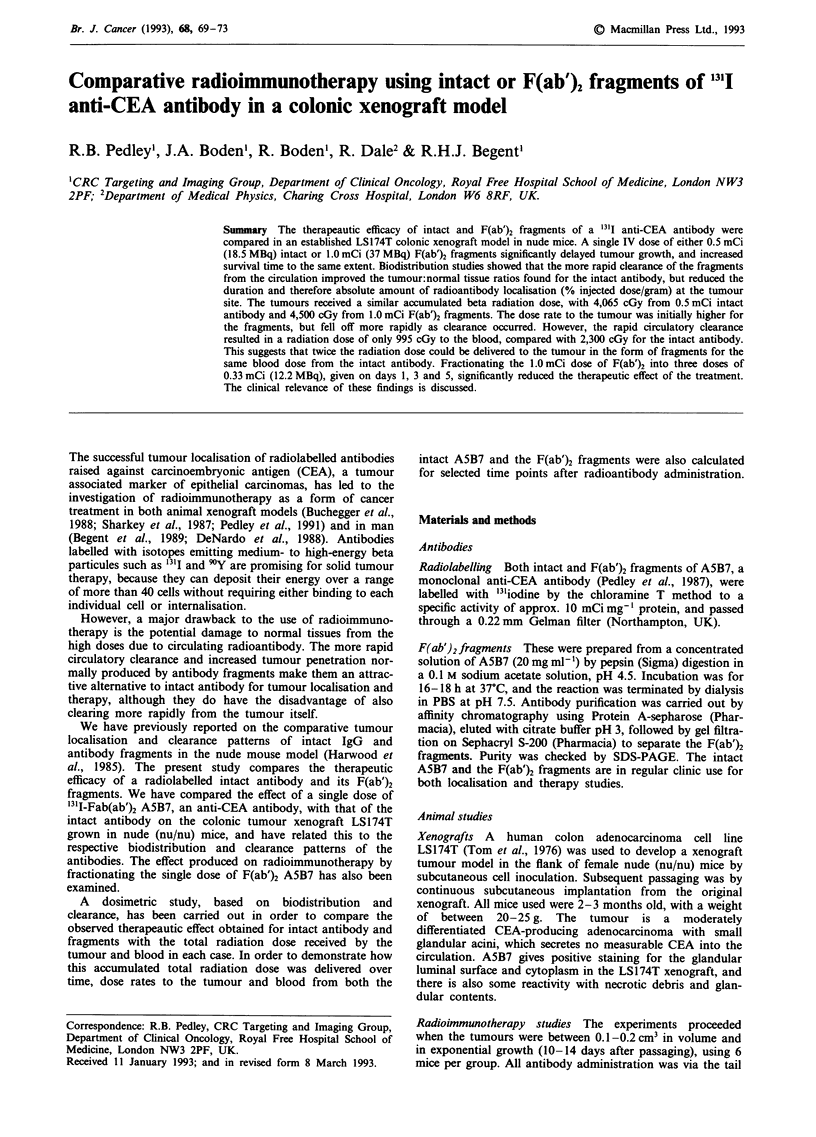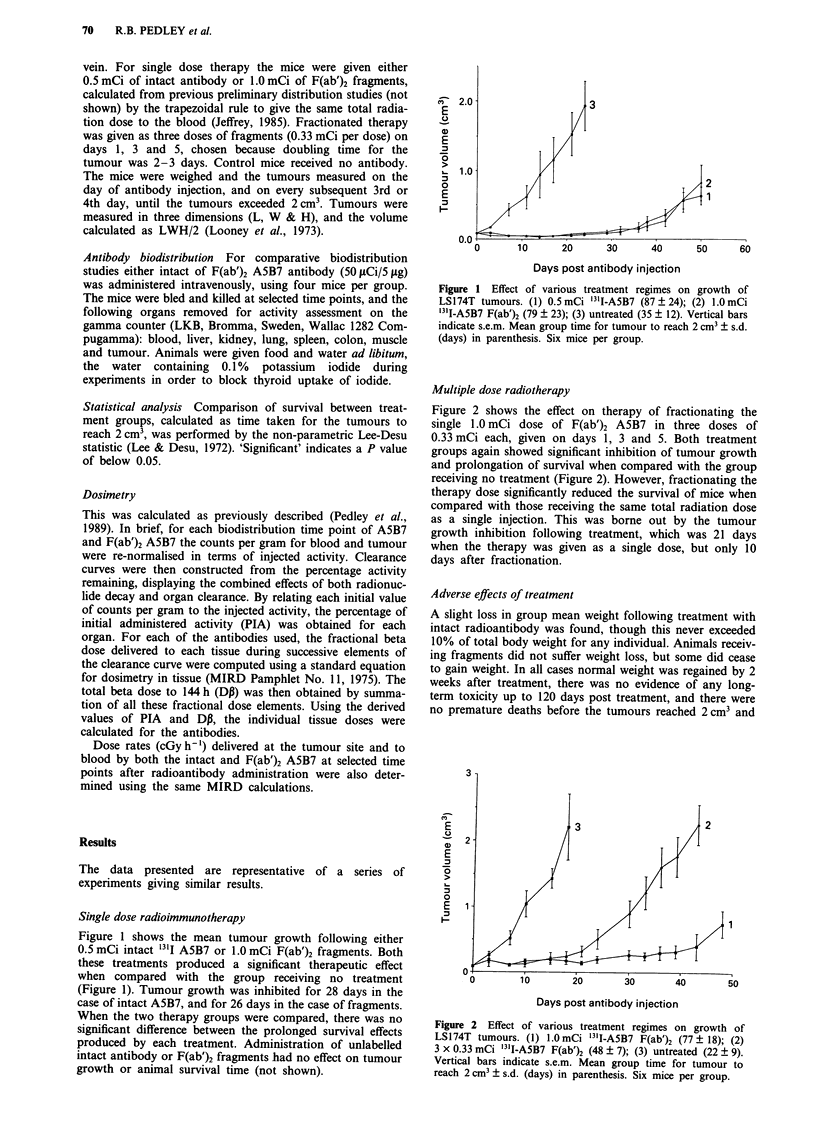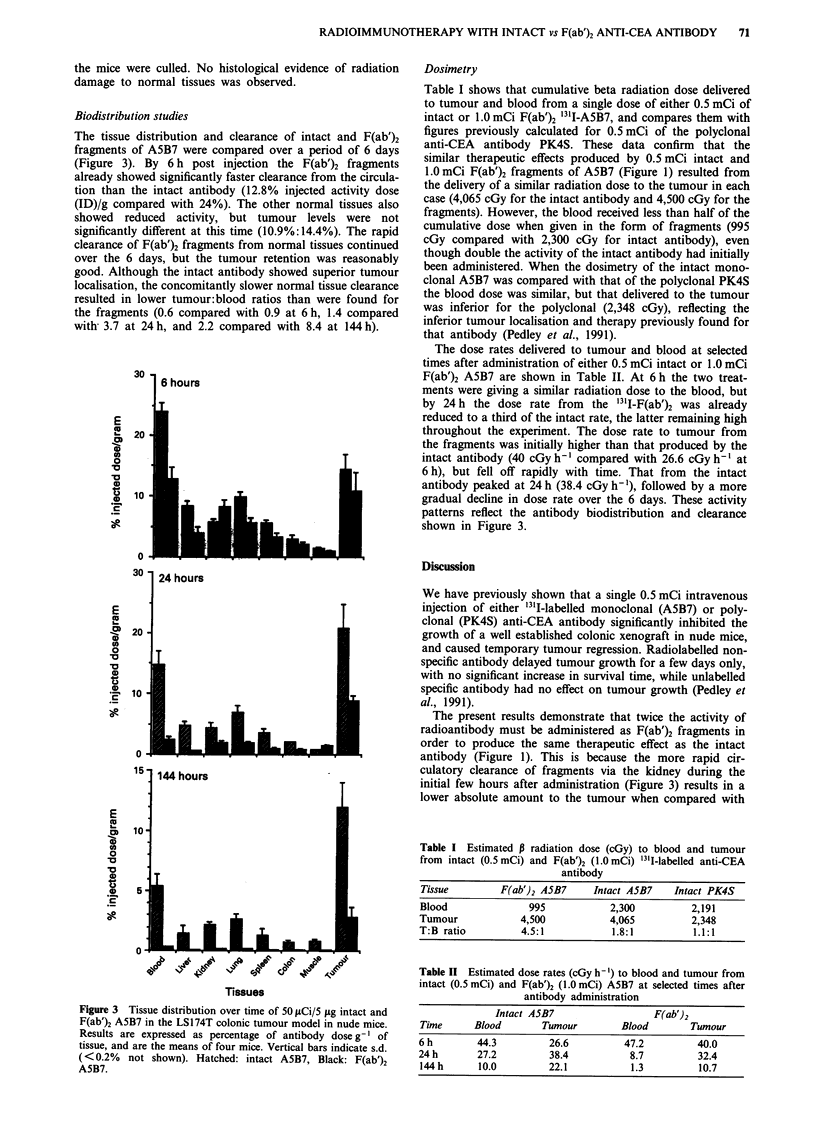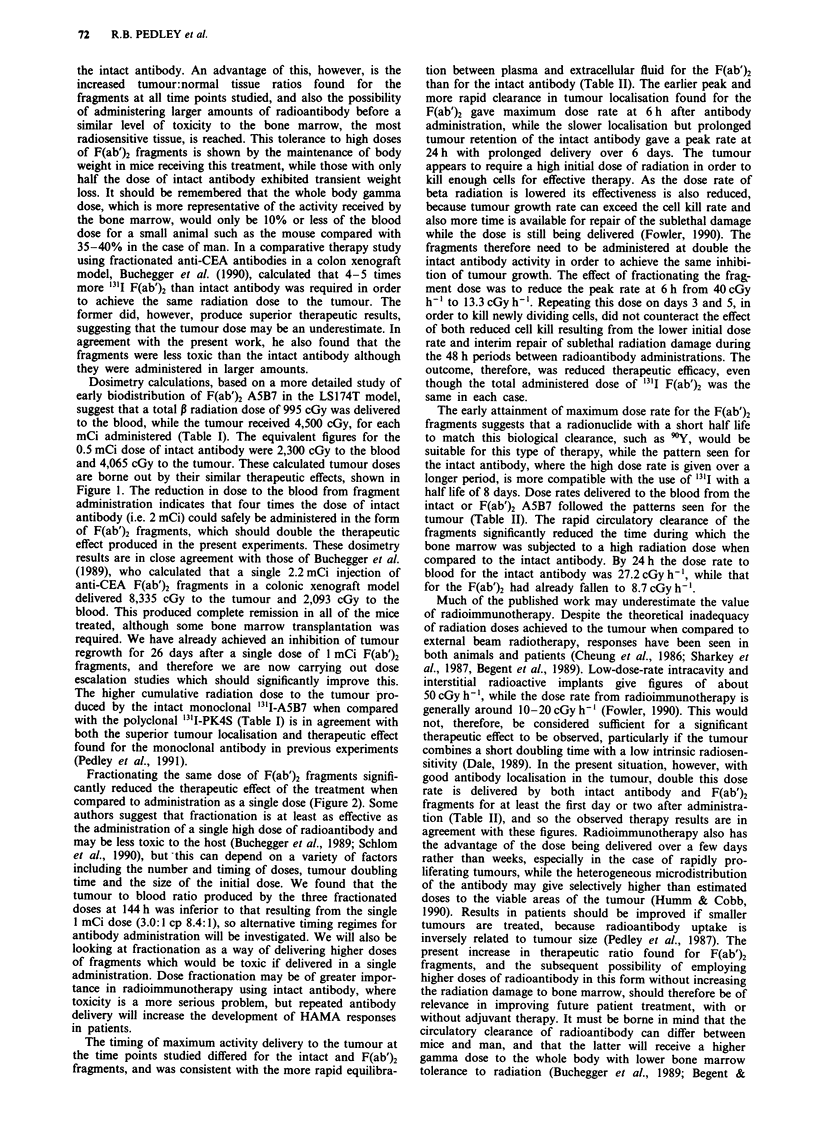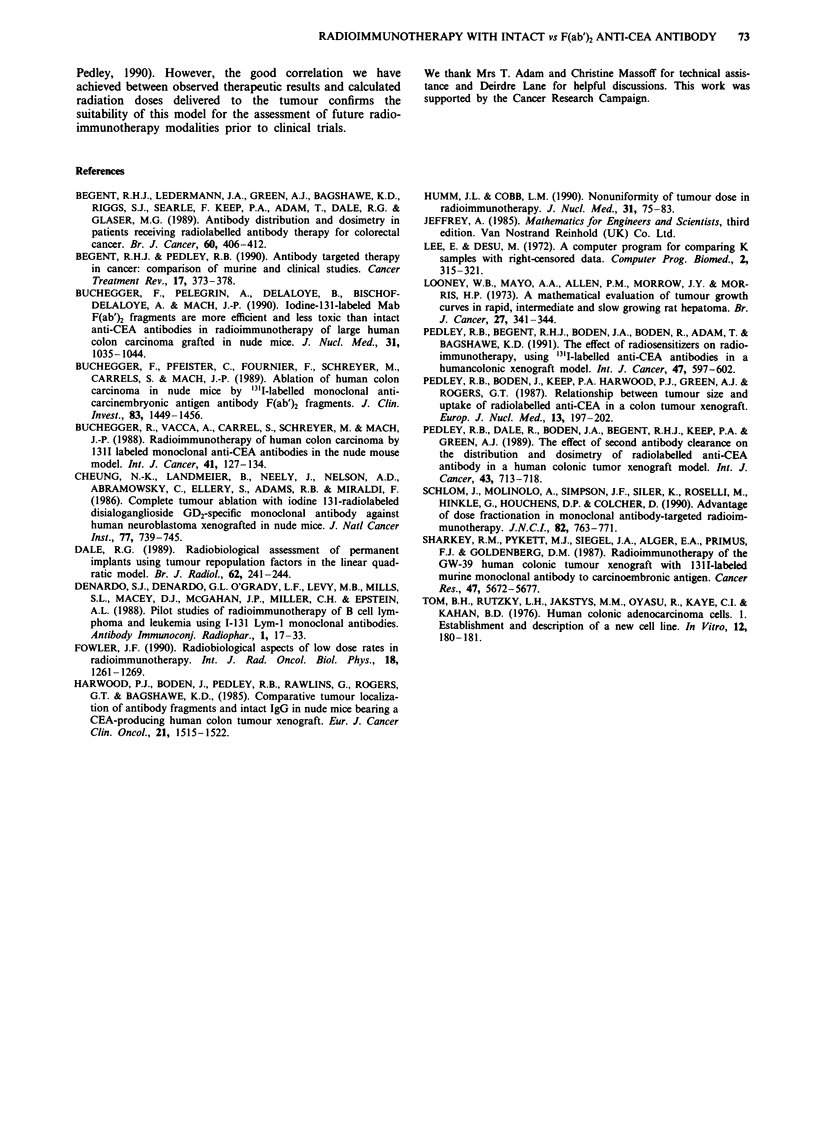# Comparative radioimmunotherapy using intact or F(ab')2 fragments of 131I anti-CEA antibody in a colonic xenograft model.

**DOI:** 10.1038/bjc.1993.288

**Published:** 1993-07

**Authors:** R. B. Pedley, J. A. Boden, R. Boden, R. Dale, R. H. Begent

**Affiliations:** Department of Clinical Oncology, Royal Free Hospital School of Medicine, London, UK.

## Abstract

The therapeutic efficacy of intact and F(ab')2 fragments of a 131I anti-CEA antibody were compared in an established LS174T colonic xenograft model in nude mice. A single IV dose of either 0.5 mCi (18.5 MBq) intact or 1.0 mCi (37 MBq) F(ab')2 fragments significantly delayed tumour growth, and increased survival time to the same extent. Biodistribution studies showed that the more rapid clearance of the fragments from the circulation improved the tumour: normal tissue ratios found for the intact antibody, but reduced the duration and therefore absolute amount of radioantibody localisation (% injected dose/gram) at the tumour site. The tumours received a similar accumulated beta radiation dose, with 4,065 cGy from 0.5 mCi intact antibody and 4,500 cGy from 1.0 mCi F(ab')2 fragments. The dose rate to the tumour was initially higher for the fragments, but fell off more rapidly as clearance occurred. However, the rapid circulatory clearance resulted in a radiation dose of only 995 cGy to the blood, compared with 2,300 cGy for the intact antibody. This suggests that twice the radiation dose could be delivered to the tumour in the form of fragments for the same blood dose from the intact antibody. Fractionating the 1.0 mCi dose of F(ab')2 into three doses of 0.33 mCi (12.2 MBq), given on days 1, 3 and 5, significantly reduced the therapeutic effect of the treatment. The clinical relevance of these findings is discussed.


Br. J. Cancer (1993), 68, 69–73 © Macmillan Press Ltd., 1993

### Comparative radioimmunotherapy using intact or $F(ab')_2$ fragments of $^{131}I$ anti-CEA antibody in a colonic xenograft model

R.B. Pedley[1], J.A. Boden[1], R. Boden[1], R. Dale[2] & R.H.J. Begent[1]

[1]*CRC Targeting and Imaging Group, Department of Clinical Oncology, Royal Free Hospital School of Medicine, London NW3 2PF;* [2]*Department of Medical Physics, Charing Cross Hospital, London W6 8RF, UK.*

**Summary** The therapeautic efficacy of intact and $F(ab')_2$ fragments of a $^{131}I$ anti-CEA antibody were compared in an established LS174T colonic xenograft model in nude mice. A single IV dose of either 0.5 mCi (18.5 MBq) intact or 1.0 mCi (37 MBq) $F(ab')_2$ fragments significantly delayed tumour growth, and increased survival time to the same extent. Biodistribution studies showed that the more rapid clearance of the fragments from the circulation improved the tumour:normal tissue ratios found for the intact antibody, but reduced the duration and therefore absolute amount of radioantibody localisation (% injected dose/gram) at the tumour site. The tumours received a similar accumulated beta radiation dose, with 4,065 cGy from 0.5 mCi intact antibody and 4,500 cGy from 1.0 mCi $F(ab')_2$ fragments. The dose rate to the tumour was initially higher for the fragments, but fell off more rapidly as clearance occurred. However, the rapid circulatory clearance resulted in a radiation dose of only 995 cGy to the blood, compared with 2,300 cGy for the intact antibody. This suggests that twice the radiation dose could be delivered to the tumour in the form of fragments for the same blood dose from the intact antibody. Fractionating the 1.0 mCi dose of $F(ab')_2$ into three doses of 0.33 mCi (12.2 MBq), given on days 1, 3 and 5, significantly reduced the therapeutic effect of the treatment. The clinical relevance of these findings is discussed.

The successful tumour localisation of radiolabelled antibodies raised against carcinoembryonic antigen (CEA), a tumour associated marker of epithelial carcinomas, has led to the investigation of radioimmunotherapy as a form of cancer treatment in both animal xenograft models (Buchegger *et al.*, 1988; Sharkey *et al.*, 1987; Pedley *et al.*, 1991) and in man (Begent *et al.*, 1989; DeNardo *et al.*, 1988). Antibodies labelled with isotopes emitting medium- to high-energy beta particules such as $^{131}I$ and $^{90}Y$ are promising for solid tumour therapy, because they can deposit their energy over a range of more than 40 cells without requiring either binding to each individual cell or internalisation.

However, a major drawback to the use of radioimmunotherapy is the potential damage to normal tissues from the high doses due to circulating radioantibody. The more rapid circulatory clearance and increased tumour penetration normally produced by antibody fragments make them an attractive alternative to intact antibody for tumour localisation and therapy, although they do have the disadvantage of also clearing more rapidly from the tumour itself.

We have previously reported on the comparative tumour localisation and clearance patterns of intact IgG and antibody fragments in the nude mouse model (Harwood *et al.*, 1985). The present study compares the therapeutic efficacy of a radiolabelled intact antibody and its $F(ab')_2$ fragments. We have compared the effect of a single dose of $^{131}I$-$Fab(ab')_2$ A5B7, an anti-CEA antibody, with that of the intact antibody on the colonic tumour xenograft LS174T grown in nude (nu/nu) mice, and have related this to the respective biodistribution and clearance patterns of the antibodies. The effect produced on radioimmunotherapy by fractionating the single dose of $F(ab')_2$ A5B7 has also been examined.

A dosimetric study, based on biodistribution and clearance, has been carried out in order to compare the observed therapeautic effect obtained for intact antibody and fragments with the total radiation dose received by the tumour and blood in each case. In order to demonstrate how this accumulated total radiation dose was delivered over time, dose rates to the tumour and blood from both the intact A5B7 and the $F(ab')_2$ fragments were also calculated for selected time points after radioantibody administration.

### Materials and methods

### *Antibodies*

*Radiolabelling* Both intact and $F(ab')_2$ fragments of A5B7, a monoclonal anti-CEA antibody (Pedley *et al.*, 1987), were labelled with $^{131}$iodine by the chloramine T method to a specific activity of approx. 10 mCi $mg^{-1}$ protein, and passed through a 0.22 mm Gelman filter (Northampton, UK).

*$F(ab')_2$ fragments* These were prepared from a concentrated solution of A5B7 (20 mg $ml^{-1}$) by pepsin (Sigma) digestion in a 0.1 M sodium acetate solution, pH 4.5. Incubation was for 16–18 h at 37°C, and the reaction was terminated by dialysis in PBS at pH 7.5. Antibody purification was carried out by affinity chromatography using Protein A-sepharose (Pharmacia), eluted with citrate buffer pH 3, followed by gel filtration on Sephacryl S-200 (Pharmacia) to separate the $F(ab')_2$ fragments. Purity was checked by SDS-PAGE. The intact A5B7 and the $F(ab')_2$ fragments are in regular clinic use for both localisation and therapy studies.

### *Animal studies*

*Xenografts* A human colon adenocarcinoma cell line LS174T (Tom *et al.*, 1976) was used to develop a xenograft tumour model in the flank of female nude (nu/nu) mice by subcutaneous cell inoculation. Subsequent passaging was by continuous subcutaneous implantation from the original xenograft. All mice used were 2–3 months old, with a weight of between 20–25 g. The tumour is a moderately differentiated CEA-producing adenocarcinoma with small glandular acini, which secretes no measurable CEA into the circulation. A5B7 gives positive staining for the glandular luminal surface and cytoplasm in the LS174T xenograft, and there is also some reactivity with necrotic debris and glandular contents.

*Radioimmunotherapy studies* The experiments proceeded when the tumours were between 0.1–0.2 $cm^3$ in volume and in exponential growth (10–14 days after passaging), using 6 mice per group. All antibody administration was via the tail

Correspondence: R.B. Pedley, CRC Targeting and Imaging Group, Department of Clinical Oncology, Royal Free Hospital School of Medicine, London NW3 2PF, UK.
Received 11 January 1993; and in revised form 8 March 1993.

vein. For single dose therapy the mice were given either 0.5 mCi of intact antibody or 1.0 mCi of $F(ab')_2$ fragments, calculated from previous preliminary distribution studies (not shown) by the trapezoidal rule to give the same total radiation dose to the blood (Jeffrey, 1985). Fractionated therapy was given as three doses of fragments (0.33 mCi per dose) on days 1, 3 and 5, chosen because doubling time for the tumour was 2–3 days. Control mice received no antibody. The mice were weighed and the tumours measured on the day of antibody injection, and on every subsequent 3rd or 4th day, until the tumours exceeded 2 $cm^3$. Tumours were measured in three dimensions (L, W & H), and the volume calculated as LWH/2 (Looney et al., 1973).

*Antibody biodistribution* For comparative biodistribution studies either intact of $F(ab')_2$ A5B7 antibody (50 $\mu$Ci/5 $\mu$g) was administered intravenously, using four mice per group. The mice were bled and killed at selected time points, and the following organs removed for activity assessment on the gamma counter (LKB, Bromma, Sweden, Wallac 1282 Compugamma): blood, liver, kidney, lung, spleen, colon, muscle and tumour. Animals were given food and water *ad libitum*, the water containing 0.1% potassium iodide during experiments in order to block thyroid uptake of iodide.

*Statistical analysis* Comparison of survival between treatment groups, calculated as time taken for the tumours to reach 2 $cm^3$, was performed by the non-parametric Lee-Desu statistic (Lee & Desu, 1972). 'Significant' indicates a $P$ value of below 0.05.

*Dosimetry*

This was calculated as previously described (Pedley et al., 1989). In brief, for each biodistribution time point of A5B7 and $F(ab')_2$ A5B7 the counts per gram for blood and tumour were re-normalised in terms of injected activity. Clearance curves were then constructed from the percentage activity remaining, displaying the combined effects of both radionuclide decay and organ clearance. By relating each initial value of counts per gram to the injected activity, the percentage of initial administered activity (PIA) was obtained for each organ. For each of the antibodies used, the fractional beta dose delivered to each tissue during successive elements of the clearance curve were computed using a standard equation for dosimetry in tissue (MIRD Pamphlet No. 11, 1975). The total beta dose to 144 h ($D\beta$) was then obtained by summation of all these fractional dose elements. Using the derived values of PIA and $D\beta$, the individual tissue doses were calculated for the antibodies.

Dose rates ($cGy\ h^{-1}$) delivered at the tumour site and to blood by both the intact and $F(ab')_2$ A5B7 at selected time points after radioantibody administration were also determined using the same MIRD calculations.

### Results

The data presented are representative of a series of experiments giving similar results.

*Single dose radioimmunotherapy*

Figure 1 shows the mean tumour growth following either 0.5 mCi intact $^{131}I$ A5B7 or 1.0 mCi $F(ab')_2$ fragments. Both these treatments produced a significant therapeutic effect when compared with the group receiving no treatment (Figure 1). Tumour growth was inhibited for 28 days in the case of intact A5B7, and for 26 days in the case of fragments. When the two therapy groups were compared, there was no significant difference between the prolonged survival effects produced by each treatment. Administration of unlabelled intact antibody or $F(ab')_2$ fragments had no effect on tumour growth or animal survival time (not shown).

**Figure 1** Effect of various treatment regimes on growth of LS174T tumours. (1) 0.5 mCi $^{131}$I-A5B7 (87 ± 24); (2) 1.0 mCi $^{131}$I-A5B7 $F(ab')_2$ (79 ± 23); (3) untreated (35 ± 12). Vertical bars indicate s.e.m. Mean group time for tumour to reach 2 $cm^3$ ± s.d. (days) in parenthesis. Six mice per group.

*Multiple dose radiotherapy*

Figure 2 shows the effect on therapy of fractionating the single 1.0 mCi dose of $F(ab')_2$ A5B7 in three doses of 0.33 mCi each, given on days 1, 3 and 5. Both treatment groups again showed significant inhibition of tumour growth and prolongation of survival when compared with the group receiving no treatment (Figure 2). However, fractionating the therapy dose significantly reduced the survival of mice when compared with those receiving the same total radiation dose as a single injection. This was borne out by the tumour growth inhibition following treatment, which was 21 days when the therapy was given as a single dose, but only 10 days after fractionation.

*Adverse effects of treatment*

A slight loss in group mean weight following treatment with intact radioantibody was found, though this never exceeded 10% of total body weight for any individual. Animals receiving fragments did not suffer weight loss, but some did cease to gain weight. In all cases normal weight was regained by 2 weeks after treatment, there was no evidence of any long-term toxicity up to 120 days post treatment, and there were no premature deaths before the tumours reached 2 $cm^3$ and

**Figure 2** Effect of various treatment regimes on growth of LS174T tumours. (1) 1.0 mCi $^{131}$I-A5B7 $F(ab')_2$ (77 ± 18); (2) 3 × 0.33 mCi $^{131}$I-A5B7 $F(ab')_2$ (48 ± 7); (3) untreated (22 ± 9). Vertical bars indicate s.e.m. Mean group time for tumour to reach 2 $cm^3$ ± s.d. (days) in parenthesis. Six mice per group.

the mice were culled. No histological evidence of radiation damage to normal tissues was observed.

*Biodistribution studies*

The tissue distribution and clearance of intact and F(ab')$_2$ fragments of A5B7 were compared over a period of 6 days (Figure 3). By 6 h post injection the F(ab')$_2$ fragments already showed significantly faster clearance from the circulation than the intact antibody (12.8% injected activity dose (ID)/g compared with 24%). The other normal tissues also showed reduced activity, but tumour levels were not significantly different at this time (10.9%:14.4%). The rapid clearance of F(ab')$_2$ fragments from normal tissues continued over the 6 days, but the tumour retention was reasonably good. Although the intact antibody showed superior tumour localisation, the concomitantly slower normal tissue clearance resulted in lower tumour:blood ratios than were found for the fragments (0.6 compared with 0.9 at 6 h, 1.4 compared with 3.7 at 24 h, and 2.2 compared with 8.4 at 144 h).

**Figure 3** Tissue distribution over time of 50 $\mu$Ci/5 $\mu$g intact and F(ab')$_2$ A5B7 in the LS174T colonic tumour model in nude mice. Results are expressed as percentage of antibody dose g$^{-1}$ of tissue, and are the means of four mice. Vertical bars indicate s.d. (<0.2% not shown). Hatched: intact A5B7, Black: F(ab')$_2$ A5B7.

*Dosimetry*

Table I shows that cumulative beta radiation dose delivered to tumour and blood from a single dose of either 0.5 mCi of intact or 1.0 mCi F(ab')$_2$ $^{131}$I-A5B7, and compares them with figures previously calculated for 0.5 mCi of the polyclonal anti-CEA antibody PK4S. These data confirm that the similar therapeutic effects produced by 0.5 mCi intact and 1.0 mCi F(ab')$_2$ fragments of A5B7 (Figure 1) resulted from the delivery of a similar radiation dose to the tumour in each case (4,065 cGy for the intact antibody and 4,500 cGy for the fragments). However, the blood received less than half of the cumulative dose when given in the form of fragments (995 cGy compared with 2,300 cGy for intact antibody), even though double the activity of the intact antibody had initially been administered. When the dosimetry of the intact monoclonal A5B7 was compared with that of the polyclonal PK4S the blood dose was similar, but that delivered to the tumour was inferior for the polyclonal (2,348 cGy), reflecting the inferior tumour localisation and therapy previously found for that antibody (Pedley *et al.*, 1991).

The dose rates delivered to tumour and blood at selected times after administration of either 0.5 mCi intact or 1.0 mCi F(ab')$_2$ A5B7 are shown in Table II. At 6 h the two treatments were giving a similar radiation dose to the blood, but by 24 h the dose rate from the $^{131}$I-F(ab')$_2$ was already reduced to a third of the intact rate, the latter remaining high throughout the experiment. The dose rate to tumour from the fragments was initially higher than that produced by the intact antibody (40 cGy h$^{-1}$ compared with 26.6 cGy h$^{-1}$ at 6 h), but fell off rapidly with time. That from the intact antibody peaked at 24 h (38.4 cGy h$^{-1}$), followed by a more gradual decline in dose rate over the 6 days. These activity patterns reflect the antibody biodistribution and clearance shown in Figure 3.

### Discussion

We have previously shown that a single 0.5 mCi intravenous injection of either $^{131}$I-labelled monoclonal (A5B7) or polyclonal (PK4S) anti-CEA antibody significantly inhibited the growth of a well established colonic xenograft in nude mice, and caused temporary tumour regression. Radiolabelled non-specific antibody delayed tumour growth for a few days only, with no significant increase in survival time, while unlabelled specific antibody had no effect on tumour growth (Pedley *et al.*, 1991).

The present results demonstrate that twice the activity of radioantibody must be administered as F(ab')$_2$ fragments in order to produce the same therapeutic effect as the intact antibody (Figure 1). This is because the more rapid circulatory clearance of fragments via the kidney during the initial few hours after administration (Figure 3) results in a lower absolute amount to the tumour when compared with

**Table I** Estimated $\beta$ radiation dose (cGy) to blood and tumour from intact (0.5 mCi) and F(ab')$_2$ (1.0 mCi) $^{131}$I-labelled anti-CEA antibody

| *Tissue* | *F(ab')$_2$ A5B7* | *Intact A5B7* | *Intact PK4S* |
|---|---|---|---|
| Blood | 995 | 2,300 | 2,191 |
| Tumour | 4,500 | 4,065 | 2,348 |
| T:B ratio | 4.5:1 | 1.8:1 | 1.1:1 |

**Table II** Estimated dose rates (cGy h$^{-1}$) to blood and tumour from intact (0.5 mCi) and F(ab')$_2$ (1.0 mCi) A5B7 at selected times after antibody administration

| | *Intact A5B7* | | *F(ab')$_2$* | |
|---|---|---|---|---|
| *Time* | *Blood* | *Tumour* | *Blood* | *Tumour* |
| 6 h | 44.3 | 26.6 | 47.2 | 40.0 |
| 24 h | 27.2 | 38.4 | 8.7 | 32.4 |
| 144 h | 10.0 | 22.1 | 1.3 | 10.7 |

the intact antibody. An advantage of this, however, is the increased tumour:normal tissue ratios found for the fragments at all time points studied, and also the possibility of administering larger amounts of radioantibody before a similar level of toxicity to the bone marrow, the most radiosensitive tissue, is reached. This tolerance to high doses of $F(ab')_2$ fragments is shown by the maintenance of body weight in mice receiving this treatment, while those with only half the dose of intact antibody exhibited transient weight loss. It should be remembered that the whole body gamma dose, which is more representative of the activity received by the bone marrow, would only be 10% or less of the blood dose for a small animal such as the mouse compared with 35–40% in the case of man. In a comparative therapy study using fractionated anti-CEA antibodies in a colon xenograft model, Buchegger *et al.* (1990), calculated that 4–5 times more $^{131}I$ $F(ab')_2$ than intact antibody was required in order to achieve the same radiation dose to the tumour. The former did, however, produce superior therapeutic results, suggesting that the tumour dose may be an underestimate. In agreement with the present work, he also found that the fragments were less toxic than the intact antibody although they were administered in larger amounts.

Dosimetry calculations, based on a more detailed study of early biodistribution of $F(ab')_2$ A5B7 in the LS174T model, suggest that a total $\beta$ radiation dose of 995 cGy was delivered to the blood, while the tumour received 4,500 cGy, for each mCi administered (Table I). The equivalent figures for the 0.5 mCi dose of intact antibody were 2,300 cGy to the blood and 4,065 cGy to the tumour. These calculated tumour doses are borne out by their similar therapeutic effects, shown in Figure 1. The reduction in dose to the blood from fragment administration indicates that four times the dose of intact antibody (i.e. 2 mCi) could safely be administered in the form of $F(ab')_2$ fragments, which should double the therapeutic effect produced in the present experiments. These dosimetry results are in close agreement with those of Buchegger *et al.* (1989), who calculated that a single 2.2 mCi injection of anti-CEA $F(ab')_2$ fragments in a colonic xenograft model delivered 8,335 cGy to the tumour and 2,093 cGy to the blood. This produced complete remission in all of the mice treated, although some bone marrow transplantation was required. We have already achieved an inhibition of tumour regrowth for 26 days after a single dose of 1 mCi $F(ab')_2$ fragments, and therefore we are now carrying out dose escalation studies which should significantly improve this. The higher cumulative radiation dose to the tumour produced by the intact monoclonal $^{131}I$-A5B7 when compared with the polyclonal $^{131}I$-PK4S (Table I) is in agreement with both the superior tumour localisation and therapeutic effect found for the monoclonal antibody in previous experiments (Pedley *et al.*, 1991).

Fractionating the same dose of $F(ab')_2$ fragments significantly reduced the therapeutic effect of the treatment when compared to administration as a single dose (Figure 2). Some authors suggest that fractionation is at least as effective as the administration of a single high dose of radioantibody and may be less toxic to the host (Buchegger *et al.*, 1989; Schlom *et al.*, 1990), but this can depend on a variety of factors including the number and timing of doses, tumour doubling time and the size of the initial dose. We found that the tumour to blood ratio produced by the three fractionated doses at 144 h was inferior to that resulting from the single 1 mCi dose (3.0:1 cp 8.4:1), so alternative timing regimes for antibody administration will be investigated. We will also be looking at fractionation as a way of delivering higher doses of fragments which would be toxic if delivered in a single administration. Dose fractionation may be of greater importance in radioimmunotherapy using intact antibody, where toxicity is a more serious problem, but repeated antibody delivery will increase the development of HAMA responses in patients.

The timing of maximum activity delivery to the tumour at the time points studied differed for the intact and $F(ab')_2$ fragments, and was consistent with the more rapid equilibration between plasma and extracellular fluid for the $F(ab')_2$ than for the intact antibody (Table II). The earlier peak and more rapid clearance in tumour localisation found for the $F(ab')_2$ gave maximum dose rate at 6 h after antibody administration, while the slower localisation but prolonged tumour retention of the intact antibody gave a peak rate at 24 h with prolonged delivery over 6 days. The tumour appears to require a high initial dose of radiation in order to kill enough cells for effective therapy. As the dose rate of beta radiation is lowered its effectiveness is also reduced, because tumour growth rate can exceed the cell kill rate and also more time is available for repair of the sublethal damage while the dose is still being delivered (Fowler, 1990). The fragments therefore need to be administered at double the intact antibody activity in order to achieve the same inhibition of tumour growth. The effect of fractionating the fragment dose was to reduce the peak rate at 6 h from 40 cGy $h^{-1}$ to 13.3 cGy $h^{-1}$. Repeating this dose on days 3 and 5, in order to kill newly dividing cells, did not counteract the effect of both reduced cell kill resulting from the lower initial dose rate and interim repair of sublethal radiation damage during the 48 h periods between radioantibody administrations. The outcome, therefore, was reduced therapeutic efficacy, even though the total administered dose of $^{131}I$ $F(ab')_2$ was the same in each case.

The early attainment of maximum dose rate for the $F(ab')_2$ fragments suggests that a radionuclide with a short half life to match this biological clearance, such as $^{90}Y$, would be suitable for this type of therapy, while the pattern seen for the intact antibody, where the high dose rate is given over a longer period, is more compatible with the use of $^{131}I$ with a half life of 8 days. Dose rates delivered to the blood from the intact or $F(ab')_2$ A5B7 followed the patterns seen for the tumour (Table II). The rapid circulatory clearance of the fragments significantly reduced the time during which the bone marrow was subjected to a high radiation dose when compared to the intact antibody. By 24 h the dose rate to blood for the intact antibody was 27.2 cGy $h^{-1}$, while that for the $F(ab')_2$ had already fallen to 8.7 cGy $h^{-1}$.

Much of the published work may underestimate the value of radioimmunotherapy. Despite the theoretical inadequacy of radiation doses achieved to the tumour when compared to external beam radiotherapy, responses have been seen in both animals and patients (Cheung *et al.*, 1986; Sharkey *et al.*, 1987, Begent *et al.*, 1989). Low-dose-rate intracavity and interstitial radioactive implants give figures of about 50 cGy $h^{-1}$, while the dose rate from radioimmunotherapy is generally around 10–20 cGy $h^{-1}$ (Fowler, 1990). This would not, therefore, be considered sufficient for a significant therapeutic effect to be observed, particularly if the tumour combines a short doubling time with a low intrinsic radiosensitivity (Dale, 1989). In the present situation, however, with good antibody localisation in the tumour, double this dose rate is delivered by both intact antibody and $F(ab')_2$ fragments for at least the first day or two after administration (Table II), and so the observed therapy results are in agreement with these figures. Radioimmunotherapy also has the advantage of the dose being delivered over a few days rather than weeks, especially in the case of rapidly proliferating tumours, while the heterogeneous microdistribution of the antibody may give selectively higher than estimated doses to the viable areas of the tumour (Humm & Cobb, 1990). Results in patients should be improved if smaller tumours are treated, because radioantibody uptake is inversely related to tumour size (Pedley *et al.*, 1987). The present increase in therapeutic ratio found for $F(ab')_2$ fragments, and the subsequent possibility of employing higher doses of radioantibody in this form without increasing the radiation damage to bone marrow, should therefore be of relevance in improving future patient treatment, with or without adjuvant therapy. It must be borne in mind that the circulatory clearance of radioantibody can differ between mice and man, and that the latter will receive a higher gamma dose to the whole body with lower bone marrow tolerance to radiation (Buchegger *et al.*, 1989; Begent &

Pedley, 1990). However, the good correlation we have achieved between observed therapeutic results and calculated radiation doses delivered to the tumour confirms the suitability of this model for the assessment of future radioimmunotherapy modalities prior to clinical trials.

We thank Mrs T. Adam and Christine Massoff for technical assistance and Deirdre Lane for helpful discussions. This work was supported by the Cancer Research Campaign.

### References

BEGENT, R.H.J., LEDERMANN, J.A., GREEN, A.J., BAGSHAWE, K.D., RIGGS, S.J., SEARLE, F. KEEP, P.A., ADAM, T., DALE, R.G. & GLASER, M.G. (1989). Antibody distribution and dosimetry in patients receiving radiolabelled antibody therapy for colorectal cancer. *Br. J. Cancer*, **60**, 406-412.

BEGENT, R.H.J. & PEDLEY, R.B. (1990). Antibody targeted therapy in cancer: comparison of murine and clinical studies. *Cancer Treatment Rev.*, **17**, 373-378.

BUCHEGGER, F., PELEGRIN, A., DELALOYE, B., BISCHOF-DELALOYE, A. & MACH, J.-P. (1990). Iodine-131-labeled Mab F(ab')$_2$ fragments are more efficient and less toxic than intact anti-CEA antibodies in radioimmunotherapy of large human colon carcinoma grafted in nude mice. *J. Nucl. Med.*, **31**, 1035-1044.

BUCHEGGER, F., PFEISTER, C., FOURNIER, F., SCHREYER, M., CARRELS, S. & MACH, J.-P. (1989). Ablation of human colon carcinoma in nude mice by $^{131}$I-labelled monoclonal anti-carcinembryonic antigen antibody F(ab')$_2$ fragments. *J. Clin. Invest.*, **83**, 1449-1456.

BUCHEGGER, R., VACCA, A., CARREL, S., SCHREYER, M. & MACH, J.-P. (1988). Radioimmunotherapy of human colon carcinoma by 131I labeled monoclonal anti-CEA antibodies in the nude mouse model. *Int. J. Cancer*, **41**, 127-134.

CHEUNG, N.-K., LANDMEIER, B., NEELY, J., NELSON, A.D., ABRAMOWSKY, C., ELLERY, S., ADAMS, R.B. & MIRALDI, F. (1986). Complete tumour ablation with iodine 131-radiolabeled disialoganglioside GD$_2$-specific monoclonal antibody against human neuroblastoma xenografted in nude mice. *J. Natl Cancer Inst.*, **77**, 739-745.

DALE, R.G. (1989). Radiobiological assessment of permanent implants using tumour repopulation factors in the linear quadratic model. *Br. J. Radiol.*, **62**, 241-244.

DENARDO, S.J., DENARDO, G.L. O'GRADY, L.F., LEVY, M.B., MILLS, S.L., MACEY, D.J., MCGAHAN, J.P., MILLER, C.H. & EPSTEIN, A.L. (1988). Pilot studies of radioimmunotherapy of B cell lymphoma and leukemia using I-131 Lym-1 monoclonal antibodies. *Antibody Immunoconj. Radiophar.*, **1**, 17-33.

FOWLER, J.F. (1990). Radiobiological aspects of low dose rates in radioimmunotherapy. *Int. J. Rad. Oncol. Biol. Phys.*, **18**, 1261-1269.

HARWOOD, P.J., BODEN, J., PEDLEY, R.B., RAWLINS, G., ROGERS, G.T. & BAGSHAWE, K.D., (1985). Comparative tumour localization of antibody fragments and intact IgG in nude mice bearing a CEA-producing human colon tumour xenograft. *Eur. J. Cancer Clin. Oncol.*, **21**, 1515-1522.

HUMM, J.L. & COBB, L.M. (1990). Nonuniformity of tumour dose in radioimmunotherapy. *J. Nucl. Med.*, **31**, 75-83.

JEFFREY, A. (1985). *Mathematics for Engineers and Scientists*, third edition. Van Nostrand Reinhold (UK) Co. Ltd.

LEE, E. & DESU, M. (1972). A computer program for comparing K samples with right-censored data. *Computer Prog. Biomed.*, **2**, 315-321.

LOONEY, W.B., MAYO, A.A., ALLEN, P.M., MORROW, J.Y. & MORRIS, H.P. (1973). A mathematical evaluation of tumour growth curves in rapid, intermediate and slow growing rat hepatoma. *Br. J. Cancer*, **27**, 341-344.

PEDLEY, R.B., BEGENT, R.H.J., BODEN, J.A., BODEN, R., ADAM, T. & BAGSHAWE, K.D. (1991). The effect of radiosensitizers on radioimmunotherapy, using $^{131}$I-labelled anti-CEA antibodies in a humancolonic xenograft model. *Int. J. Cancer*, **47**, 597-602.

PEDLEY, R.B., BODEN, J., KEEP, P.A. HARWOOD, P.J., GREEN, A.J. & ROGERS, G.T. (1987). Relationship between tumour size and uptake of radiolabelled anti-CEA in a colon tumour xenograft. *Europ. J. Nucl. Med.*, **13**, 197-202.

PEDLEY, R.B., DALE, R., BODEN, J.A., BEGENT, R.H.J., KEEP, P.A. & GREEN, A.J. (1989). The effect of second antibody clearance on the distribution and dosimetry of radiolabelled anti-CEA antibody in a human colonic tumor xenograft model. *Int. J. Cancer*, **43**, 713-718.

SCHLOM, J., MOLINOLO, A., SIMPSON, J.F., SILER, K., ROSELLI, M., HINKLE, G., HOUCHENS, D.P. & COLCHER, D. (1990). Advantage of dose fractionation in monoclonal antibody-targeted radioimmunotherapy. *J.N.C.I.*, **82**, 763-771.

SHARKEY, R.M., PYKETT, M.J., SIEGEL, J.A., ALGER, E.A., PRIMUS, F.J. & GOLDENBERG, D.M. (1987). Radioimmunotherapy of the GW-39 human colonic tumour xenograft with 131I-labeled murine monoclonal antibody to carcinoembronic antigen. *Cancer Res.*, **47**, 5672-5677.

TOM, B.H., RUTZKY, L.H., JAKSTYS, M.M., OYASU, R., KAYE, C.I. & KAHAN, B.D. (1976). Human colonic adenocarcinoma cells. 1. Establishment and description of a new cell line. *In Vitro*, **12**, 180-181.